# Modeling Challenges of Ebola Virus–Host Dynamics during Infection and Treatment

**DOI:** 10.3390/v12010106

**Published:** 2020-01-16

**Authors:** Daniel S. Chertow, Louis Shekhtman, Yoav Lurie, Richard T. Davey, Theo Heller, Harel Dahari

**Affiliations:** 1 Critical Care Medicine Department, National Institutes of Health Clinical Center, Laboratory of Immunoregulation, National Institute of Allergy and Infectious Diseases, Bethesda, MD 20892, USA; 2 The Program for Experimental and Theoretical Modeling, Division of Hepatology, Department of Medicine, Loyola University Medical Center, Maywood, IL 60153, USA; lsheks@gmail.com (L.S.); hdahari@luc.edu (H.D.); 3 Network Science Institute, Northeastern University, Boston, MA 02115, USA; 4 Liver Unit, Shaare Zedek Medical Center and the Hebrew University of Jerusalem, Jerusalem 9103102, Israel; 5 Division of Intramural Research, National Institute of Allergy and Infectious Diseases, National Institutes of Health, Bethesda, MD 20892, USA; rdavey@niaid.nih.gov; 6 Translational Hepatology Unit, Liver Diseases Branch, National Institute of Diabetes and Digestive and Kidney Diseases, National Institutes of Health, Bethesda, MD 20892, USA; theoh@intra.niddk.nih.gov

**Keywords:** Ebola virus, mathematical modeling, viral kinetics, liver

## Abstract

Mathematical modeling of Ebola virus (EBOV)–host dynamics during infection and treatment in vivo is in its infancy due to few studies with frequent viral kinetic data, lack of approved antiviral therapies, and limited insight into the timing of EBOV infection of cells and tissues throughout the body. Current in-host mathematical models simplify EBOV infection by assuming a single homogeneous compartment of infection. In particular, a recent modeling study assumed the liver as the largest solid organ targeted by EBOV infection and predicted that nearly all cells become refractory to infection within seven days of initial infection without antiviral treatment. We compared our observations of EBOV kinetics in multiple anatomic compartments and hepatocellular injury in a critically ill patient with Ebola virus disease (EVD) with this model’s predictions. We also explored the model’s predictions, with and without antiviral therapy, by recapitulating the model using published inputs and assumptions. Our findings highlight the challenges of modeling EBOV–host dynamics and therapeutic efficacy and emphasize the need for iterative interdisciplinary efforts to refine mathematical models that might advance understanding of EVD pathogenesis and treatment.

## 1. Introduction

Additional strategies to prevent and treat Ebola virus disease (EVD) are urgently needed, as mortality remains unacceptably high and no proven-effective therapies are presently available [1]. In support of preclinical animal models, simple mathematical models that account for Ebola virus (EBOV) titer kinetics, might provide insight into the timing of when EBOV therapeutics or prophylactics might be effective [2]. More complex models that account for EBOV viral titer kinetics, susceptible cells, EBOV-infected cells, and immune response dynamics might advance understanding of EVD pathogenesis and assist in prioritization of therapies for rigorous evaluation in randomized clinical trials (RCTs) [3]. Recently, Madelain et al. [4] developed a single-anatomic compartment immuno-pathogenesis mathematical model to provide insights into EBOV–host dynamics in nonhuman primates and predict effectiveness of favipiravir and remdesivir, both viral RNA polymerase inhibitors, for post exposure prophylaxis and treatment of EVD in humans.

We discuss the assumption of a single anatomic compartment of EBOV infection in the models described by Madelain et al. [4] and Martyushev et al. [3], and the selection of the liver as the largest solid organ targeted by EBOV by Madelain et al. We compare assumptions from these models with our observations in a critically ill patient with EVD who was cared for at the National Institutes of Health (NIH) Clinical Center who developed severe meningoencephalitis and multiorgan failure and received supportive care alone without experimental therapy [5].

To better understand EBOV–host interactions and conditions of antiviral efficacy predicted by the Madelain et al. model, we replicated their model using published inputs and assumptions. Our findings highlight the challenges of modeling EBOV–host interactions and therapeutic efficacy and emphasize the need for iterative interdisciplinary efforts to refine mathematical models to further advance understanding of EVD pathogenesis and treatment.

## 2. Material and Methods

### 2.1. Patients

We previously measured daily viral RNA levels in serum [5], and intermittently in semen [6] of a critically ill patient with EVD who was cared for at the NIH Clinical Center during acute illness and recovery. In the current study viral RNA levels in tracheal aspirate on days 17 to 19 and in axillary sweat on days 21, 24, and 25 post-symptom onset were measured. EZ-1 reverse-transcription quantitative polymerase chain reaction (RT-qPCR) testing was used to quantify viral RNA as previously described [6,7]. Daily aspartate aminotransferase (AST) and alanine aminotransferase (ALT) levels were measured in whole blood at the point of care during acute illness using the Piccolo Xpress (Abaxis, Union City, CA, USA) blood chemistry analyzer and expressed as a ratio of AST to ALT. All assays performed fell within permissible usage in the original patient consent under NIH IRB protocol #15-I-0083.

### 2.2. Mathematical Modeling

We reproduced the model described in Figure 3 in Madelain et al. [4] using the parameter values outlined in Table 1 [4] using Berkeley Madonna (version 9.0). The reproduced equations are provided in the Supplementary Material.

## 3. Results

**Figure 1:**
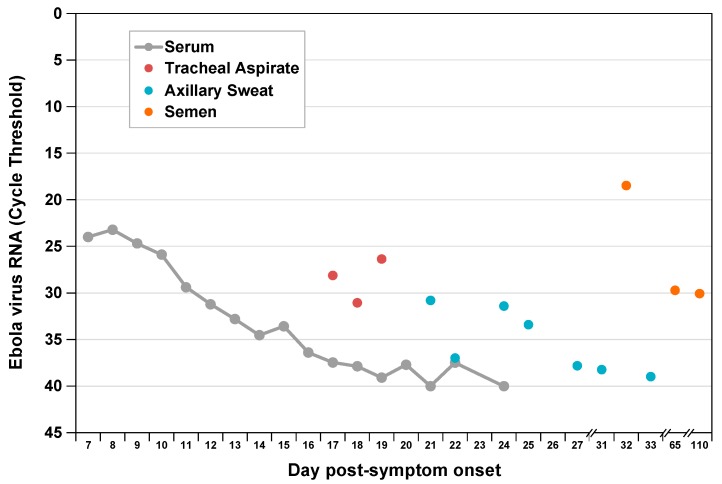
Ebola virus RNA levels by compartment during acute and convalescent illness. We measured viral RNA extracted from multiple sample types by EZ-1 quantitative reverse-transcription polymerase chain reaction assay as previously described [6]. Previously published serum and semen data are included for comparison [5,6].

**Figure 2:**
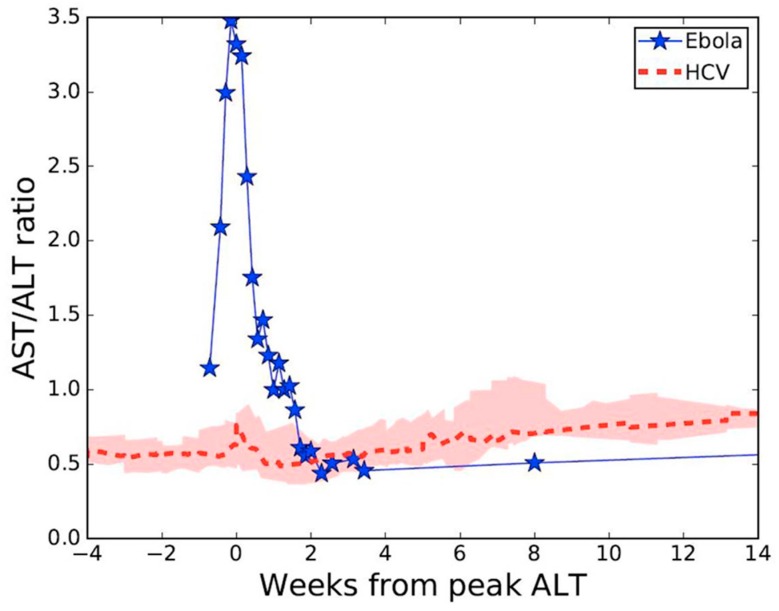
Aspartate and alanine aminotransferase (AST/ALT) ratio kinetics during acute Ebola virus (*n* = 1) [5], or acute hepatitis C virus (*n* = 28) [8,9] infections. Pink shaded region represents first and third AST/ALT ratio quartiles.

Viral RNA levels in tracheal aspirate on days 17 to 19 and in axillary sweat on days 21, 24, and 25 of post-symptom onset were significantly elevated relative to previously reported levels in serum [5] at matched time points (Figure 1). High concentration replicating virus was detected in semen during recovery [6]. At day 8 post-symptom onset in our patient, we observed a peak AST to ALT ratio of 3.5 compared with a ratio <1.0 in a cohort of patients with acute hepatitis C infection [8,9] (Figure 2), where viral replication and cellular injury are limited to the liver.

**Figure 3:**
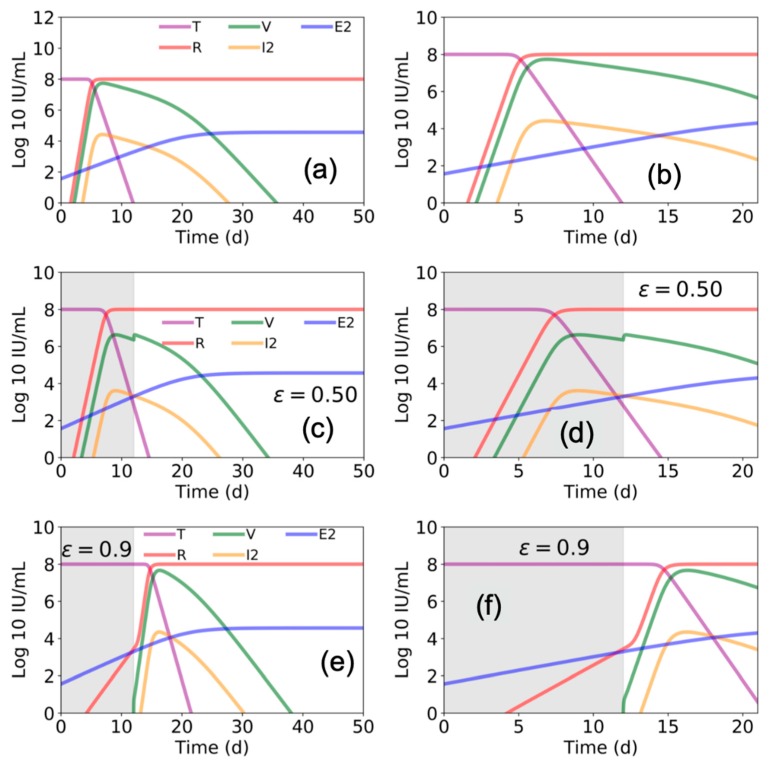
Estimated Ebola virus–host dynamics with and without antiviral treatment. Using parameter values presented in Figure 3 and Table 1 in Madelain et al. [4], we plot the values of target cells (T), viral load (V), refractory cells (R), productive infected cells (I2), and EBOV specific T cells (E2) with (**a**,**b**) zero antiviral efficacy (ε = 0), (**c**,**d**) with 50% efficacy (ε = 0.5), and (**e**,**f**) with 90% antiviral efficacy (ε = 0.9). Estimates over 50 days are shown in (**a**,**c**,**e**) and a zoom of the first 21 days are shown in (**b**,**d**,**f**). Gray shaded areas indicate duration of antiviral treatment.

We ran the model described by Madelain et al. using the best estimated parameter space (reported in Table 1 in Madelain et al. [4]) to gain further understanding of the suggested interplay among EBOV, the liver, and immune response. We found that Madelain’s model suggests that without antiviral treatment (ε = 0), within 7 days post infection ~99% of pre-infection liver (or target) cells become refractory (R) to EBOV infection (Figure 3a,b). Accordingly, viral load (V) and productive EBOV-infected cells (I2) peak at day ~7 post infection followed by viral decline.

To advance understanding of the model’s predicated effects of antiviral treatment in blocking viral production, we simulated the model assuming a fixed drug efficacy of ε = 0.5 or ε = 0.9 (as predicted for favipiravir or remdesivir, respectively) from days 0 to day 12 post infection (i.e., the duration of antiviral treatment in animals in Madelain et al. [4]). Our simulations agreed with the reported predictions of Madelain et al. for ε = 0.5 (Figure 3c,d). However, under higher efficacy antiviral treatment (ε = 0.9), the model predicted a delay in timing when ~99% of pre-infection liver cells became refractory with a higher peak in V and I2 (Figure 3e,f) compared with lower efficacy antiviral treatment (ε = 0.5) (Figure 3c,d) when treatment was stopped at day 12 post infection.

**Figure 4:**
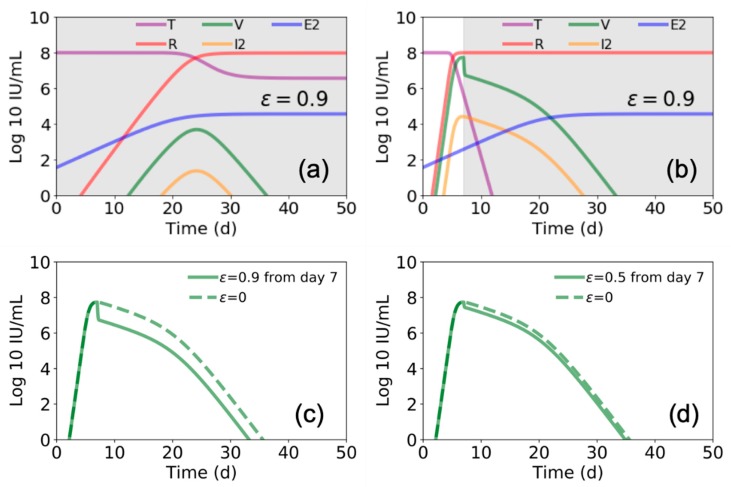
Estimated Ebola virus–host dynamics with antiviral treatment for different periods. In (**a**) and (**b**) we again use the parameter values presented in Figure 3 and Table 1 [4], and plot the values of target cells (T), viral load (V), refractory cells (R), productive infected cells (I2), and EBOV specific T cells (E2). In (**a**) we show this for treatment ε = 0.9 beginning at day 0 and continuing through day 50, while in (**b**) we show for treatment beginning at day 7 and continuing through day 50 (gray shaded areas indicate duration of antiviral treatment). In (**c**,**d**) we compare the viral load for the case of starting treatment at day 5 and continuing through day 50 for (**c**) ε = 0.9 and (**d**) ε = 0.5.

We further found that the model by Madelain et al. predicts that if remdesivir is initiated from the time of infection and continues for an extended interval, a longer viral ramp-up with a lower peak (Figure 4a) and 100% survival is expected [4]. However, if remdesivir is initiated after peak viral load (i.e., ~7 days post infection), there is a limited effect on viral load (compare Figure 4b–d with Figure 3a,b) and a significant increase in predicted mortality, suggesting a very narrow therapeutic window for remdesivir.

## 4. Discussion

The assumption made by Madelain et al. [4] and Martyushev et al. [3] of one compartment of EBOV infection and replication that represents multiple organs that are infected at the same time is counter to significant evidence that EBOV infects cells and tissues throughout the body in a nonhomogeneous fashion [10]. EBOV initially infects immune cells within the subcutaneous or submucosal compartments which drain to adjacent lymph nodes and support high-level viral replication during an average six-day incubation period [11]. Following symptom onset, EBOV is then widely disseminated in the blood infecting the spleen, liver, kidney, and multiple other organs throughout the body. Our observations in a critically ill patient with EVD cared for at the NIH without experimental therapy, support variability in viral kinetics across multiple anatomic compartments and are consistent with findings of others who described the variability in clearance of EBOV RNA or live virus from multiple body fluids including saliva, tears, sweat, breast milk, vaginal fluid, urine, stool, cerebrospinal fluid, aqueous humor, and semen [12]. Improved understanding of the kinetics of EBOV infection in tissues and cells throughout the body will allow for the development of more biologically accurate mathematical models of EBOV–host dynamics.

Madelain et al. [4] assumed the liver as the largest solid organ targeted by EBOV infection in their model, and therefore set the initial number of target cells as 10^8^ cells/mL, as a proxy of the liver size in nonhuman primates. We observed a peak AST/ALT ratio of 3.5 in our patient at the time of peak hepatocellular injury, compared with a ratio <1.0 among a cohort of patients with acute HCV infection. The difference in these ratios suggests that the predominant proportion of cellular injury in our patient was derived from organs other than the liver, such as muscle or kidney [13]. Which of these compartments is the predominant compartment of EBOV infection, and what proportion of cellular injury is attributable to direct virally-mediated versus indirect cellular injury, remains undetermined. Our observations are consistent with that of others where an AST/ALT ratio >2 is the predominant pattern observed in humans and nonhuman primates infected with EBOV [14,15] and where rhabdomyolysis and acute kidney injury frequently complicate severe disease [16]. Improved understanding of the contribution of virally-mediated versus indirect cell death in multiple body compartments will aid in the development of more accurate and predictive mathematical models of EVD.

Postmortem histopathologic analysis of human and nonhuman primate liver specimens supports direct infection and necrosis of hepatocytes in a focal to widespread fashion [10,17]. Degenerate or necrotic hepatocytes frequently include intracytoplasmic viral inclusion bodies or stain positive for EBOV antigens by immunohistochemical staining, supporting a significant contribution of direct virally-mediated hepatocellular injury and death. Madelain et al.’s model predicts that ~99% of hepatocytes, as a surrogate for all susceptible cells in the body, become refractory to infection by day ~7 post infection without antiviral therapy, and thus nearly all liver cells remain uninfected without antiviral therapy. If this were true, then focal to widespread hepatic necrosis observed in human and nonhuman primate postmortem liver specimens must be attributable to indirect rather than direct virally-mediated death. Histopathologic liver findings in human and nonhuman primates, however, support that direct virally-mediated hepatocellular injury significantly contributes to overall hepatocellular death.

During the 2013–2016 EBOV outbreak in West Africa, therapeutic efficacy of Z-Mapp, a cocktail of three monoclonal antibodies (mAb) targeting EBOV surface glycoprotein (GP), was evaluated in an RCT [18], although waning cases precluded a conclusive result. During the ongoing EBOV outbreak in the Democratic Republic of the Congo (DRC), Z-Mapp, Mab114, and REGN-EB3, all mAb-based therapies, and remdesivir, a viral polymerase inhibitor, were evaluated in an RCT to determine their impact on survival [19]. Trial results showed a survival benefit with REGN-EB3 and mAb114 relative to Z-Mapp and remdesivir [19]. Overall, 49.7% of patients who received Z-Mapp and 53.1% of patients who received remdesivir died compared with 33.5% and 35.1% of patients who received REGN-EB3 and mAb114, respectively.

The ability to more accurately predict efficacy of experimental EVD therapies in human RCTs based on preclinical animal and mathematical models would aid in the prioritization of competing EVD therapies and trial designs. Mathematical models that closely reflect complex pathogen–host interactions in multiple anatomic compartments could assist in understanding the potential benefit of competing therapies with different mechanisms of action, as is the case with mAb-based versus antiviral therapies for EVD. Interestingly, our recapitulation of Madelain et al. model predicts that withdrawal of a highly-effective antiviral therapy (ε = 0.9) that starts immediately post-exposure and continues through day 12 post infection, would result in a sharp increase in susceptible cells and high viral load when treatment is stopped at day 12 (Figure 3e,f). In contrast, only a slight increase in viral load was predicted if a less effective therapy (ε = 0.5) is administered for 12 days (Figure 3c,d). While this contrast seems biologically implausible, additional animal experiments are required to support or refute this prediction. It should be noted, however, that we simulated the model of Madelaine et al. once using fixed estimated parameters provided in Table 1 [4] and that model predictions may vary with slight changes in model inputs.

Among the four experimental EVD therapies evaluated in the RCT in the DRC, early treatment compared with late treatment was associated with improved survival [19]. Consistent with findings in this RCT, recapitulation of Madelain et al. model predicts that the window of remdesivir efficacy is extremely narrow. However, mAb-based therapies have out-performed remdesivir in the DRC RCT, confirming that treatment mechanism of action in addition to timing of administration impacts outcome. Existing mathematical models of viral–host dynamics in EVD do not account for different mechanisms of action of EVD therapeutics, limiting the utility for prioritizing treatments for RTC evaluation or informing trial designs.

A significant public health challenge during the current EVD outbreak is delayed case findings and thus delayed isolation of cases, contact tracing, safe and dignified burials, ring vaccination, and medical interventions. Development of therapies that effectively reduce mortality when administered later in the disease course remains a priority. Robust mathematical models that accurately predict efficacy of treatments based upon timing of use and mechanism of action might guide the evaluation and use of these treatments in the field. However, such models must accurately reflect the complex biology and pathogenesis of natural infection. Madelain et al. [4] have developed the most comprehensive model of EBOV–host dynamics to date. Future efforts at refining EVD mathematical models should account for viral kinetics in multiple anatomic compartments and the contribution of viral versus host-mediated cellular injury and death. Understanding how these, and other important factors, vary in the setting of experimental therapies with different mechanisms of action in preclinical animal models will inform the development of robust mathematical models that hold promise to advance the treatment and prevention of severe emerging and re-emerging global infectious disease threats.

## Supplementary Materials

The following are available online at https://www.mdpi.com/1999-4915/12/1/106/s1.
